# Deep sequencing of the 16S ribosomal RNA of the neonatal oral microbiome: a comparison of breast-fed and formula-fed infants

**DOI:** 10.1038/srep38309

**Published:** 2016-12-06

**Authors:** S. S. Al-Shehri, E. L. Sweeney, D. M. Cowley, H. G. Liley, P. D. Ranasinghe, B. G. Charles, P. N. Shaw, D. Vagenas, J. A. Duley, C. L. Knox

**Affiliations:** 1 School of Pharmacy, The University of Queensland, St Lucia, 4102, Australia; 2 School of Applied Medical Sciences, Taif University, Taif, 21974, Saudi Arabia; 3 The Institute of Health and Biomedical Innovation, Faculty of Health, School of Biomedical Sciences, Queensland University of Technology, Brisbane, 4059, Australia; 4 Mater Research Institute, The University of Queensland, Woolloongabba, 4102, Australia; 5 Queensland University of Technology, Science and Engineering Faculty, School of Earth, Environmental and Biological Sciences, 4001, Australia

## Abstract

*In utero* and upon delivery, neonates are exposed to a wide array of microorganisms from various sources, including maternal bacteria. Prior studies have proposed that the mode of feeding shapes the gut microbiota and, subsequently the child’s health. However, the effect of the mode of feeding and its influence on the development of the neonatal oral microbiota in early infancy has not yet been reported. The aim of this study was to compare the oral microbiota of healthy infants that were exclusively breast-fed or formula-fed using 16S-rRNA gene sequencing. We demonstrated that the oral bacterial communities were dominated by the phylum *Firmicutes*, in both groups. There was a higher prevalence of the phylum *Bacteroidetes* in the mouths of formula-fed infants than in breast-fed infants (*p* = 0.01), but in contrast *Actinobacteria* were more prevalent in breast-fed babies; *Proteobacteria* was more prevalent in saliva of breast-fed babies than in formula-fed neonates (*p* = 0.04). We also found evidence suggesting that the oral microbiota composition changed over time, particularly *Streptococcus* species, which had an increasing trend between 4–8 weeks in both groups. This study findings confirmed that the mode of feeding influences the development of oral microbiota, and this may have implications for long-term human health.

The fetus is first exposed to bacteria *in utero*^1^, and following delivery is colonized with diverse microorganisms at different bodily sites (termed the microbiota). This microbial colonization initiates maturation of the infant immune system and alterations to the microbiota (dysbiosis) may result in illness or increased risk of infection, particularly during infancy. Within the first few hours of life, the most important sources of neonatal microbiota are derived from the mother’s vaginal, fecal and skin microbiota^2^,^3^. The presence of these microorganisms, and those from different environmental sources are likely to contribute to the development and regulation of the oral bacterial microbiota in the first few months of life^4^.

Within the neonatal oral cavity, microorganisms may be regulated by other mechanisms. In a recent study, our group discovered that neonatal saliva contains high levels of the metabolites xanthine and hypoxanthine; these are substrates of the enzyme xanthine oxidase (XO), which is highly abundant within breast milk^5^,^6^. During breast-feeding, the mixing of neonatal saliva with breast milk generates hydrogen peroxide, a reactive oxygen species (ROS) that in turn activates the ‘lactoperoxidase system’ to produce additional bactericidal ROS, as well as hypothiocyanate and nitrite. These metabolites provide a unique antibacterial activity within the neonatal mouth at a time when other immune mechanisms are not yet fully developed. Furthermore, we demonstrated that some pathogenic bacteria, including *Staphylococcus aureus* and *Salmonella* species, were preferentially inhibited by the production of ROS during *in vitro* experiments when simulated neonatal saliva and expressed human breast milk were combined with these bacteria^6^.

Breast milk has long been considered a superior food for infants, particularly as breast-feeding has been associated with a reduced incidence of infection^7^,^8^. Formula feeding has been shown to induce the most rapid weight gain in preterm infants, while breast milk has a lower protein and calorie content than formula milk it is rich in immunoglobulins, enzymes and growth factors. Recent research has demonstrated that neonatal immune mechanisms may be inadequate for regulating the gastrointestinal tract (GIT) microbiota, in particular with formula-fed babies who are at an increased risk of developing necrotising enterocolitis - the most common and lethal GIT emergency in preterm infants^9^. In contrast, preterm babies who are fed breast milk are three times less likely to develop necrotising enterocolitis than formula-fed babies^10^,^11^. Infants thus appear to rely on ‘innate’ immune mechanisms, which may be present in breast milk, to regulate their GIT microbiota until the development of the more mature cellular and antibody-mediated immune system present in children and adults^12^.

Previous studies have investigated infant microbiota, in particular the development of the infant GIT microbiota, demonstrating key differences depending on the mode of delivery^4^,^13^,^14^. More recently, differences in the oral microbiota of three-month-old infants were reported depending on the mode of feeding, by Holgerson *et al*.^15^, who found that *Lactobacillus* species were isolated from breast-fed infants but not from formula-fed infants, and there were also major differences in 14 taxa (detected by HOMIM microarray) depending on the mode of feeding^15^. Another smaller study of six infants mapped the changes in the distribution of microorganisms within the skin, oral and GIT microbiota between 8–21 days of life^16^. However, the infants enrolled in this study were low-birth weight, predominantly preterm and all of these infants were treated with antibiotics during the study period. As a result, it is not known if these findings are consistent with the development of the microbiota within a healthy, term-born population. Whilst these two studies have extended our knowledge and understanding of the infant microbiota, it remains unclear how the microbiota of the oral cavity alters over time in healthy infants in the early neonatal period.

Studies of the human microbiota have not been possible without the use of next-generation sequencing (NGS). This useful tool has allowed us to better understand the relationship between microbiota and its association to human health and disease^17^,^18^. The results obtained by molecular techniques, including NGS, are far superior to that of culture-based technologies and a large number of samples may be tested within a single assay^19^. For this current study we hypothesised that the oral microbiota of infants varies depending on the mode of feeding (i.e. breast-feeding *versus* formula-feeding) and with age. We utilised 16S rRNA gene deep sequencing to characterize the oral microbiota of healthy 4–8 week-old infants who were exclusively breast-fed or formula-fed.

## Results

### Participants for *in vivo* studies of oral bacteria

Among the 38 neonates recruited (delivered vaginally; 26 breast-fed *versus* 12 formula-fed), there were no significant differences in gestational age (*p* = 0.3), birth weight (*p* = 0.2), or age (*p* = 0.2) (Table 1). The mothers and infants enrolled in this study did not receive any antibiotics prior to or at the time of sample collection.

### Overview of 16S rRNA sequencing on breast-fed and formula-fed infants: sequences, quality analyses and taxa identified

The microbiota swabbed from the inner cheek surfaces of breast-fed and formula-fed infants (age: 4 or 6 or 8 weeks), yielded 496,303 curated high quality reads of 250–550 bp with an average length of 492.97 ± 8.33 bp (see Supplementary Info Table 1).

**Figure 1:**
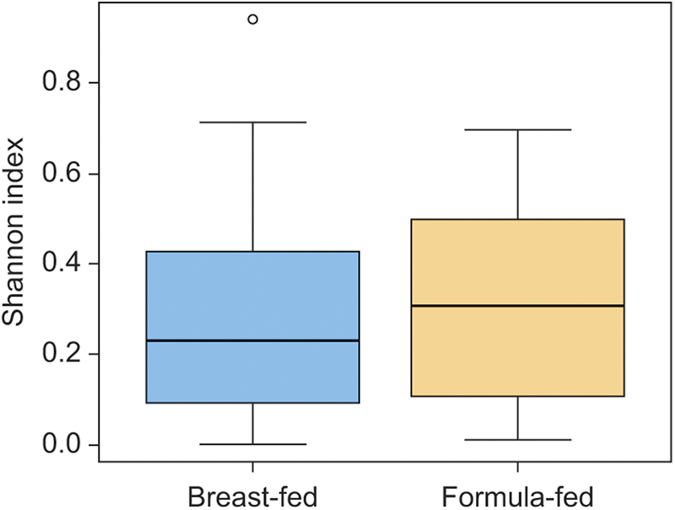
Oral bacterial community richness analyses. Alpha diversity measurements using a Shannon index analysis indicated samples with higher and lower Genus diversity in the mouths of breast-fed and formula-fed neonates. The depth of sequence reads was drawn against the richness of OTU clusters at different taxonomic assignments.

Following removal of low quality sequences, which comprised 11.5% of the original dataset, the bacterial richness was assessed as the Shannon index, an indicator of mean richness in bacterial diversity. There were similarities among breast-fed and formula-fed infants, with formula-fed infants demonstrating a slightly higher (mean) diversity within their oral microbiota (Fig. 1).

### Oral bacterial community structure revealed differences in the relative abundance of bacteria phyla among breast-fed and formula-fed infants

**Figure 2:**
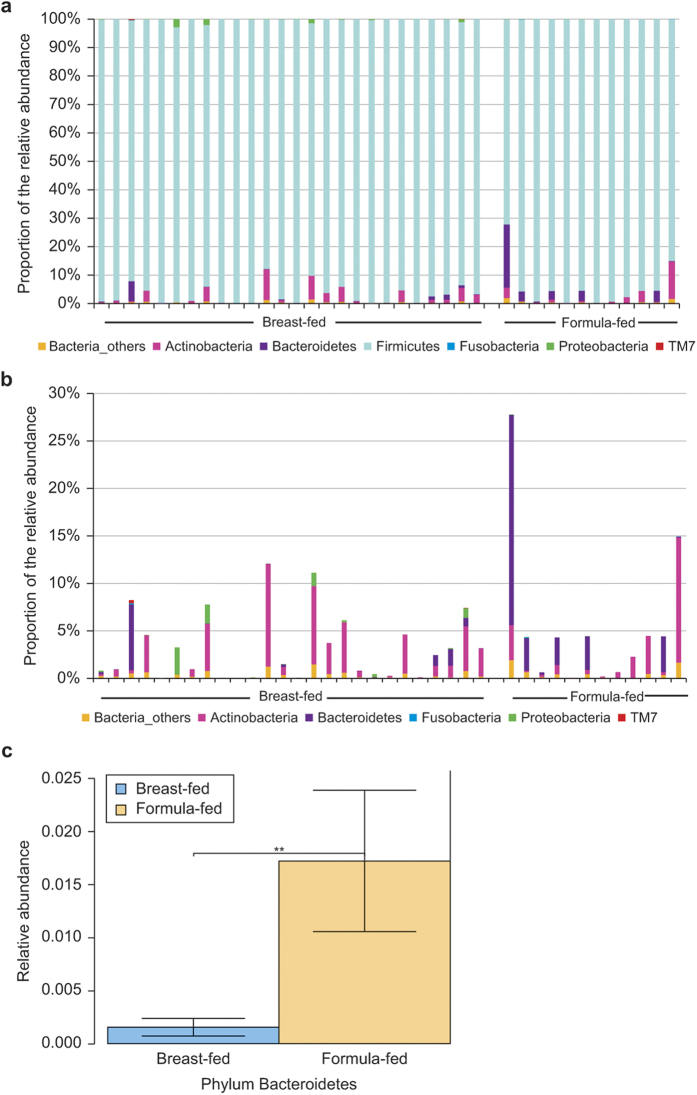
Proportion of the relative abundance of the major oral bacterial phyla. Taxa were detected by 16S rRNA analysis using Roche-454 GS FLX deep sequencing. Each column within the bar graph represents the percentages of the bacterial OTUs detected within each infant oral swab sample. (**a**) Bar graph showing the proportion of the relative abundance (%) of the major oral phyla operational taxonomic units (OTUs) for breast-fed samples (n = 26) and formula-fed samples (n = 12). (**b**) Bar graph showing the relative abundance (%) of oral phyla excluding the *Firmicutes,* of both breast-fed and formula-fed oral samples. There were key differences in the abundance of some phyla among breast-fed and formula-fed infants. (**c**) Relative abundance of the Phylum *Bacteroidetes* in oral swab samples from breast-fed and formula-fed infants (**p = 0.01).

Analyses of operational taxonomic units (OTUs) from the oral cavity of breast-fed and formula-fed infants clustered within four main bacterial phyla: *Actinobacteria*, *Bacteroidetes*, *Firmicutes*, and *Proteobacteria*. The *Firmicutes* represented the most predominant phylum in the oral cavity of both breast-fed and formula-fed infants, accounting for (mean ± SD) 96.3% ± 0.04 of the OTUs in breast-fed infants and 95.3% ± 0.05 in formula-fed infants (*p* = 0.5) (Fig. 2a).

The relative distributions of the remaining bacterial phyla were re-analyzed after excluding the *Firmicutes,* to highlight the prevalence of the other bacterial communities present (Fig. 2b). These analyses revealed that some of the phylum-level distribution patterns were significantly different between the mouths of breast-fed and formula-fed babies. The phylum *Actinobacteria* was the second most abundant in breast-fed neonates, representing 2.1% ± 0.03 of the OTUs in oral samples, *versus* 1.6% ± 0.03 in formula-fed samples (*p* = 0.8). In contrast, the phylum *Bacteriodetes* was the next most abundant in formula-fed infants (1.8% ± 0.6 of the OTUs) compared with breast-fed neonates (0.2% ± 0.1 of the OTUs) (*p* = 0.01) (Fig. 2c.). For breast-fed neonates, the phylum *Proteobacteria* was also abundant, but the prevalence of this phylum was very low in formula-fed neonates (*p* = 0.04).

**Figure 3:**
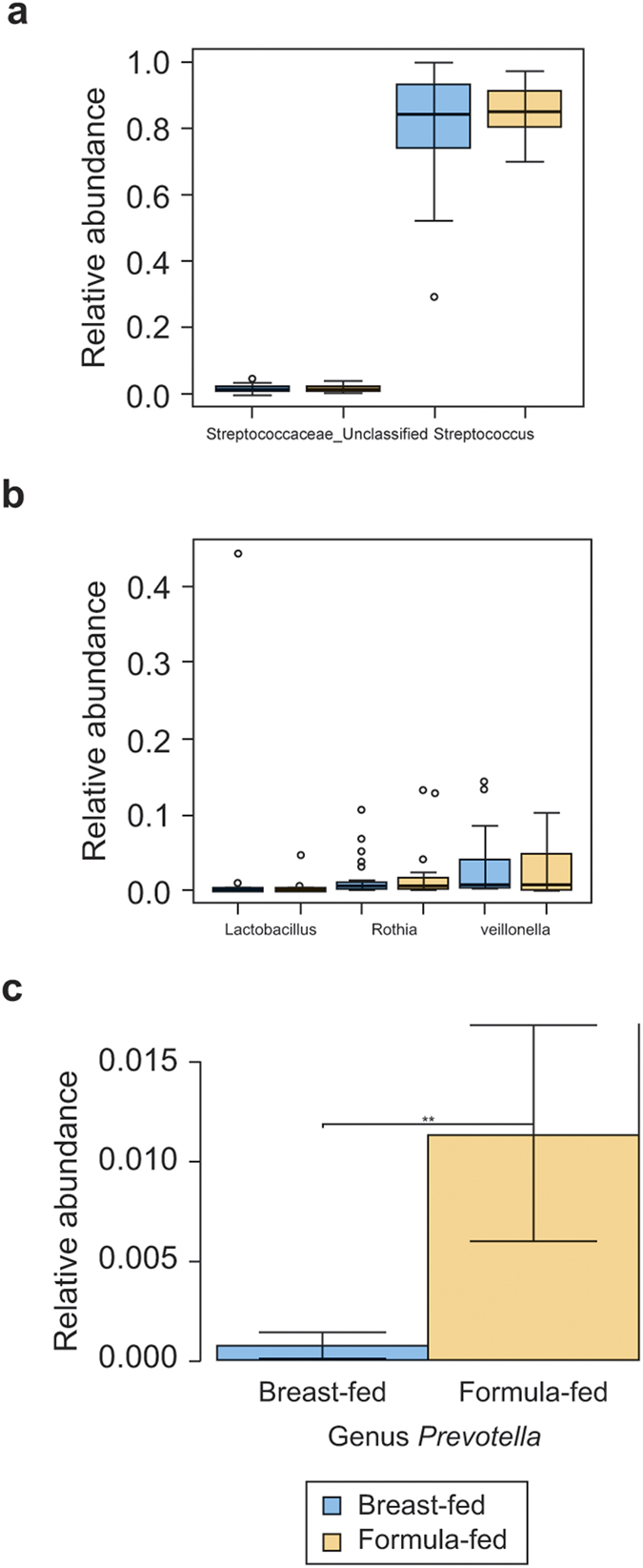
Relative abundance of 16S rRNA Genus-level sequences. (**a**) Genus level sequences were detected in oral swabs from breast-fed (red) and formula-fed (blue) infants using deep sequencing. Values are expressed as box plot showing the mean, percentiles, and SD. (**b**) The relative abundance of sequences that were classified only to the Family level ‘*Streptococcaceae*_unclassified’, compared to known *Streptococcus* spp. sequences. (**c**) The relative abundance of the major Genera sequences detected in the oral samples: *Lactobacillus* spp. (Phylum *Firmicutes*), *Rothia* spp. (Phylum *Actinobacteria*) and *Veillonella* spp. (Phylum *Firmicutes*). (**d**) *Prevotella* spp. (Phylum *Bacteroidetes*) sequences were more abundant the oral samples from formula-fed compared to breast-fed infants (*p* = 0.02).

Furthermore, the bacteria collected from the oral cavity of breast-fed and formula-fed infants were analysed for genus-level mean distributions of the three major phyla within all samples: results are shown as box plots in Fig. 3. The *Streptococcus* spp. (Phylum *Firmicutes*) was the predominant genus identified in the oral cavities of both breast-fed and formula-fed infants. Less than 1% of these reads could not be classified to the genus level (shown as family “*Streptococcaceae*_unclassified”; (Fig. 3a), which may have been as a consequence of genetic similarities in the gene portion amplified within this study. There was no significant difference in *Streptococcus* spp. prevalence between breast- and bottle-fed babies.

**Figure 4:**
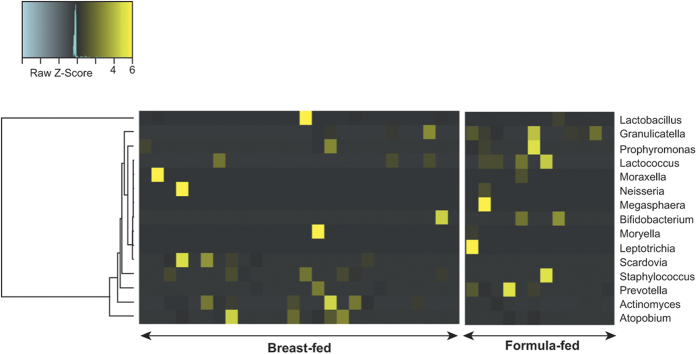
Heat map of oral bacterial community compositions within breast-fed and formula-fed infants and their hierarchical clustering. The 16S rRNA gene sequencing revealed differences in the abundance of oral bacterial communities. The bacterial genera are represented as heat maps corresponding to a phylogenetic tree. The heat map demonstrates the higher abundance of *Prevotella* spp. within the oral samples from breast-fed compared to formula-fed infants.

There were no differences in the prevalence of the genera *Lactobacillus* (*Firmicutes*)*, Rothia* (*Actinobacteria*) and *Veillonella* (*Firmicutes*) in the oral cavity of breast-fed and formula-fed infants (Fig. 3b). However, *Prevotella* spp. (Phylum *Bacteriodetes*) were significantly more abundant in the oral cavity of formula-fed infants (*p* = 0.02), when compared to breast-fed infants (Fig. 3c). The relative abundance of the bacteria shown in Fig. 3 were further plotted using a Heat Map (Fig. 4), which demonstrates the higher abundance of *Prevotella* spp. within the oral cavity of formula-fed infants when compared to breast-fed infants.

**Figure 5:**
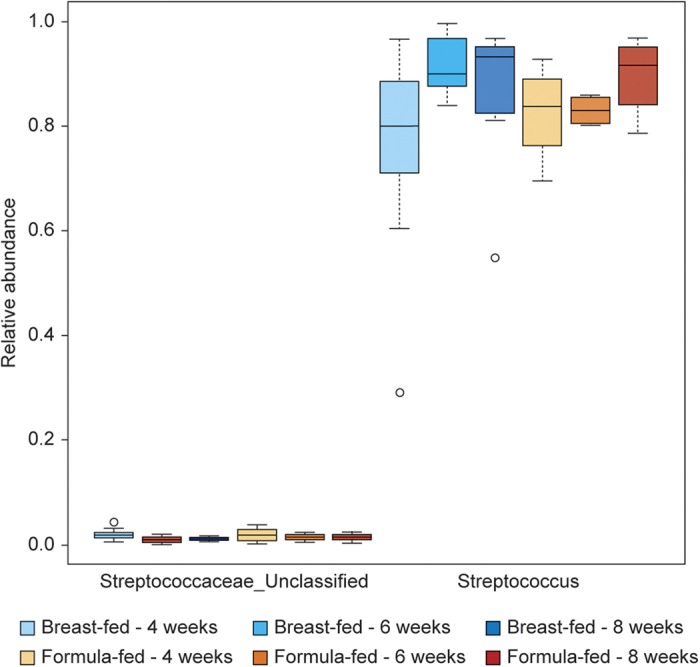
Temporal changes in streptococcal relative abundance in oral samples from breast-fed and formula-fed infants. The relative abundance of oral *Streptococcus* Genus-level sequences increased from weeks 4 to 8, for both breast-fed and formula-fed infants. Paralleling this increase, there was a decrease in the number of Genus-level sequences that could not be classified (‘*Streptococcaceae*_unclassified’) from 4, 6 and 8 weeks of life. Each box plot corresponds to a sample time point and the mode of infant feeding versus the abundance of the OTUs. Box plots showing the mean, percentiles and SD.

Interestingly, the abundance of both *Streptococcus* spp. and ‘*Streptococcaceae*_unclassified’ for each collection time cohort (4, 6, 8 weeks for both breast-fed and formula-fed infants) demonstrated apparent changes over time (Fig. 5). The analysis showed that while there was a trend of increasing relative abundance of *Streptococcus* spp. over time, there was a corresponding decrease in the relative abundance of unclassified Family *Streptococcaceae*.

**Figure 6:**
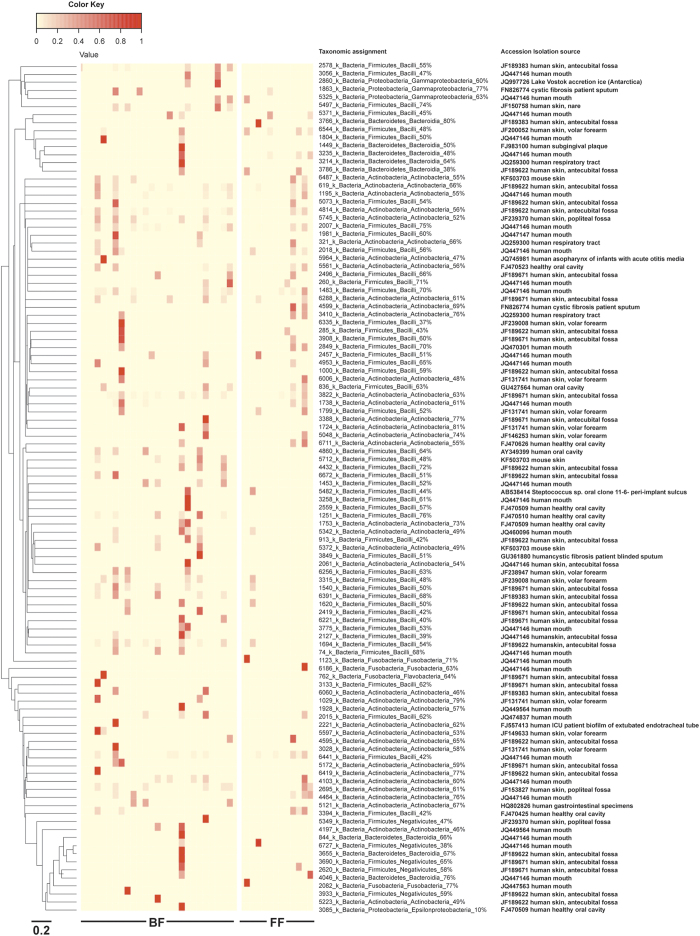
Heat map of sequences identified as ‘Bacteria_others’ within oral samples from infants. The heat map demonstrates the abundance of ‘Bacteria_others’ sequences and the taxonomic identities (phylogeny). The identities were explored using an RDP naïve Bayesian classifier and are shown at the Class level. The percentage values (taxonomic assignment) indicate the homology to bacteria classified to the Class level that have been isolated/identified previously, and the accession isolation source indicates the sites from which these closely-related bacterial DNA sequences have been previously isolated.

For those reads identified as ‘Bacteria_others’, we further investigated the closest known identities by comparing these sequence data against databases of known bacterial sequences. Figure 6 shows the accession numbers and the sources of the most closely related sequences that we were able to extract, from http://www.ncbi.nlm.nih.gov/gene/. We found that the majority of sequences of the ‘Bacteria_others’ were most closely related (10–18% homology) to bacterial sequences originally isolated from either the skin surfaces on the human arm (47%), the human mouth (40%), the human lower respiratory tract (7%), or the human upper respiratory tract (2%). Only one sequence was related closely to organisms isolated from the GIT, while four sequences were environmental isolates. Furthermore the sequences of ‘Bacteria_others’ were most closely related to the Phylum *Firmicutes* (Class *Bacilli* 45%; *Negativecutes* 5%), Phylum *Actinobacteria* (35%), Phylum *Bacteroidetes* (7.5%), Phylum *Fusobacteria* (3.75%) and Phylum *Proteobacteria* (3.75%).

## Discussion

An important priority for studying the human microbiome is to improve our knowledge and understanding of the bacteria implicated in human health and disease states. The microbiota has been suggested as a potential target for therapeutic interventions for a range of diseases^20^,^21^. While it is known that the oral microbiome of adults is relatively stable and may harbour up to 700 different bacterial species^22^, it is likely that the infant oral microbiome is more dynamic and may be influenced by a range of factors, including diet and the infant’s developing immune competency. Our current research investigated the composition of the oral bacterial microbiota in breast-fed and formula-fed infants at 4–8 weeks of life, using 16S rRNA gene deep sequencing. We demonstrated distinct differences in the oral bacterial communities associated with breast-feeding and formula-feeding.

The most abundant bacterial phylum within the oral cavity of both breast-fed and formula-fed infants was the *Firmicutes.* and this was consistent with reports of others^14^,^23^. Interestingly, *Actinobacteria* was the second most prevalent oral phylum in breast-fed babies, while the phylum *Bacteriodetes*, which includes the genus *Bacteroides,* was the second most prevalent in the mouths of formula-fed neonates but was infrequently detected in infants who were breast-fed. These findings were consistent with previous studies of infant GIT microbiota, in which *Bacteriodetes* was the phylum most commonly found in association with formula-feeding^4^ and these microorganisms were also highly abundant within the faeces of formula-fed infants^24^. In contrast, the phylum *Proteobacteria* (which include the ubiquitous GIT bacteria *Escherichia coli*) was more abundant in the mouths of breast-fed infants when compared to formula-fed infants. The *Proteobacteria* are common bacteria in the GIT of neonates^25^ and reported as the predominant phylum (65%) in human breast milk^24^. A recent study has suggested the existence of an entero-mammary pathway for the transfer of these bacteria from the GIT of the mother to the neonate^26^. We suggest therefore, that the breast-fed neonates in our current study may have acquired such *Proteobacteria* from breast-feeding; this hypothesis is further strengthened by the very low abundance of these bacteria within the oral cavity of formula-fed infants.

We also found that the genus *Prevotella* was significantly more abundant in the mouths of formula-fed infants. *Prevotella* spp. are part of the normal oral and vaginal microflora; an association has been demonstrated between the presence of *Prevotella* spp. in the mouths of mothers and the isolation of these microorganisms in their infants’ saliva^27^. Subsequent studies identified unique strains of *Prevotella* spp. within the mouths of infants’ that did not match the isolates obtained from the mother’s saliva^28^. This suggests that there may be alternative reservoirs or influences allowing these microorganisms to colonise the infant mouth: the results of our study and those of Kononen *et al*., suggested that the mode of feeding may influence the colonisation of the oral cavity with *Prevotella* spp.

While studies have demonstrated an association between infant oral and GIT microbiota in relation to mode of delivery^13^,^23^, very few studies have investigated the infant oral microbiome in relation to the mode of feeding. Holgerson *et al*.^15^ studied 73 infants at three months of age and reported differences within the oral microbiota of breast-fed versus formula–fed infants who were exclusively/partially breast-fed, or formula-fed^15^. Their results and the findings of our current study provide further confirmation that the mode of feeding affects the oral microbiota of infants from the early neonatal period (4 weeks of age) and up to 3 months of age. Human breast milk also has its own unique microbiome^29^, so breast-feeding of infants may assist in the transmission of ‘healthy’ microflora from mothers to their infants. However, it is also known that pathogenic viruses (human immunodeficiency virus, Zika virus, cytomegalovirus) and bacteria (*Candida* spp., *Klebsiella* spp., *Acinetobacter* spp. and *Staphylococcus* spp.) may be acquired from an infected mother during breast feeding, predisposing to dysbiosis and disease^30^,^31^,^32^.

Saliva and breast milk contain growth-enhancing and antibacterial factors, including amino acids, organic acids, proteins, fats, carbohydrates, enzymes, and immunoglobulins. Thus, the differences in oral microbiota reported here for breastfed *versus* formula-fed infants cannot be attributed solely to the production of ROS described in our previous study^6^, which provided *in vitro* evidence for the mixing of neonatal saliva and breast milk in the regulation of the infant oral microbiota^6^. Our present *in vivo* findings support the concept of microbial regulation according to the mode of feeding, and agree with previous reports that the neonatal microflora of the GIT differs significantly between breast-fed and formula-fed infants^4^,^33^. Our study only included infants delivered vaginally, to minimize the effect of mode of delivery on oral bacterial communities, and excluded mothers or babies who received antimicrobial treatment. Other studies have demonstrated differences for the oral microbiota of infants delivered vaginally compared with those born by Cesarean section^13^, and for infants who have been treated with antimicrobials^16^.

It was demonstrated here that the infant oral microbiota is dynamic between 4–8 weeks of age. The abundance of Family *Streptococc*a*ceae*, the most common oral bacterial family identified in both breast-fed and formula-fed infants, accounting for 55–90% of all bacteria within our study. Furthermore, the abundance of the known streptococcal genera and species appeared to increase in the mouths of both breast-fed and formula-fed babies between 4 to 8 weeks of age. This was consistent with other studies showing that *Streptococcus* spp. were abundant in the saliva of infants^14^,^34^ and their incidence increased with age^16^,^35^. One study of oral microbiota in six low-birth weight infants (five of whom were born prematurely) sampled at different ages reported that the *Streptococcus* spp. and *Staphylococcus* spp. were the most abundant genera in the mouths of all babies tested and these increased significantly between 8–21 days of life^16^.

Others studies have demonstrated a “protective” role for streptococci in the oral cavity. Hydrogen peroxide-producing streptococci were shown to inhibit the growth of pathogenic microorganisms, including the nosocomial pathogens methicillin-resistant *Staphylococcus aureus* (MRSA) and *Pseudomonas aeruginosa*^36^. It has also been shown that lactic acid bacteria isolated from breast milk were able to inhibit growth of *Enterococcus faecalis*, *Salmonella enterica* subspp. *enterica*, *Listeria monocytogenes*, *Staphylococcus aureus* and *Escherichia coli*^37^. Similarly, our recent study demonstrated that the mixing of infant saliva and breast milk during breast-feeding generates hydrogen peroxide concentrations in excess of 70 μM, which were sufficient to inhibit the *in vitro* growth of pathogenic bacteria such as *Salmonella* spp.^6^. We also found unusually high concentrations of thiocyanate in neonatal saliva: this serves as a substrate for milk lactoperoxidase, which produces bactericidal ROS and hypothiocyanate^6^. Taken together, our studies implied an important role for ROS in regulating bacteria within the infant mouth. This raises the question of whether this primal metabolic mechanism for perinatally producing oral hydrogen peroxide may be replaced during weaning and teething by hydrogen peroxide-producing oral streptococci, thus maintaining the protective role of ROS throughout infancy and childhood?

While the findings of our current study are novel, there are some limitations that must be addressed. Over 50% of total reads were identified as members of the *Streptococcaceae* family and the majority were classified to genus *Streptococcus*. Some isolates belonging to the family *Streptococcaceae* were unable to be assigned to any known genus and this is likely as a consequence of the similarities in the gene sequence targeted in this study, and is consistent with the findings of others, that the discriminatory power of the 16S rRNA gene sequences was insufficient to successfully identify clinical isolates^38^,^39^. Furthermore, we found that some 16S rRNA reads could not be assigned to any known taxonomic level beyond domain bacteria (Fig. 6). Reads belonging to ‘Bacteria_others’ were most closely related to sequences derived from human sources, e.g human forearm skin, human oral or nasal cavities and human respiratory tract. These sequences may be those of novel bacterial taxa, however we utilised stringent (85%) taxonomic classifications to identify the DNA sequences within our study, while the RDP classifier was able to provide reliable taxonomic identities at 60% taxonomic identity for fragment lengths ranging from 220 bp~550 bp. These stringent conditions, coupled with the short sequence lengths generated by the 16S rRNA gene deep sequencing, may have resulted in inability to group/identify some of these bacterial sequences to the genus level.

We anticipated a high prevalence of *Bifidobacterium* spp. within the mouths of breast-fed infants, as these bacteria are among the first anaerobic microorganisms to reach high concentrations within the GIT and are responsible for human milk oligosaccharide metabolism^40^. However, we detected these bacteria in only one breast-fed and two formula-fed infants. This may be a consequence of the aerobic/microaerophilic environment of the mouth being inappropriate for their growth, or these oral microorganisms may colonise the mouth later in infancy (>8 weeks). Perhaps unsurprisingly, we identified inter-individual variations within both the breast-fed and formula-fed groups, both at taxon and phylum levels, perhaps arising from variation between host metabolisms, or the oral niches of the infants, and/or derived from maternal/environmental contact. Nonetheless, the differences were not marked and this may serve as a hallmark of stability of the microbial community and potentially act as biomarkers for infant health.

In conclusion, this current molecular study has provided novel information about the impact of breast-feeding *versus* formula-feeding on the oral microbiota of the neonate. Differences were detected in the bacterial profiles of breast-fed and formula-fed infants, contributing to our growing understanding of the infant microbiome, as well as the factors that may influence variation within the oral microbiota. We also noted alterations in the oral microbiota over time, with a trend of increasing abundance of *Streptococcus* spp. between 4, 6 and 8 weeks of life in both breast-fed and formula-fed neonates, perhaps highlighting the dynamic importance of these bacteria within the oral cavity. This study presents preliminary data prompting further research that will be critical to our understanding of the changing role of oral microbiota during neonatal development and ontogeny of dysbiosis.

## Methods

### Subjects and specimen collection

This study was approved by the Human Research Ethics Committees of the University of Queensland (2012000449) and Mater Mother’s Hospital (2012_10LNR). The methods were conducted in accordance with the approved guidelines. Parents of the eligible infants gave informed consent for their infants to be recruited for this study. Breast-fed and formula-fed infants on antibiotics or infants whose mothers were on antibiotics before or at the time of collection, were excluded from the study. Swabs of the oral/cheek surfaces were collected from healthy, full-term, vaginally-delivered, Caucasian infants aged 4, 6 and 8 weeks who had not been fed for at least 30 min prior to specimen collection. Infants’ oral cheek swab specimens were collected at the participants’ homes from breast-fed (n = 26) and formula-fed (n = 12) infants. The inner cheek surface of each infant was swabbed gently for approximately 5 min using sterile swabs (Copan flocked swabs, Italy); sterile gloves were worn by the collector during the collection process. Three swabs were collected from each infant (at 4, 6 or 8 weeks), placed in sterile 1.5 mL Eppendorf tubes and snap-frozen on dry ice. Samples were immediately delivered to the laboratory and stored at −80 °C for further analysis. Characteristics of infants included in this study are summarized in Table 1.

### DNA extraction and purification

For total genomic DNA extraction, each cheek swab (1–2 swabs (of the total of 3 swabs) per neonate per time point) was mixed with 100 μL of 4 g/L lysozyme (20 mM Tris-HCl, pH 8.0; 2 mM EDTA; 1.2% Triton) (Sigma-Aldrich Pty Ltd, Castle Hill, NSW, Australia) and then incubated in a heating block for 35 mins at 37 °C. After incubation, 100 μL of 100 μg/mL Proteinase K (Qiagen Pty Ltd, Chadstone Centre, VIC, Australia) solution was added to the mixture and this was further incubated for 30 mins at 37 °C. Finally, 400 μL of lysis buffer was added to the mixture, which then was incubated at 56 °C for a further 10 mins. The total genomic DNA was subsequently purified using the QIAamp DNA Mini Kit (Qiagen Pty Ltd) following the manufacturer’s instructions for Spin-Column Protocol for “DNA Purification from Buccal Swabs”. The genomic DNA was eluted in 100 μL of PCR grade DNAse/RNAse-free distilled water (Gibco UltraPure^TM^, Invitrogen, Mulgrave, VIC, Australia). Concentrations and the DNA quality was confirmed using a NanoDrop ND-1000 spectrophotometer (Thermo Scientific, Scoresby, VIC, Australia) and by agarose gel electrophoresis in a 2% w/v tris borate EDTA (TBE) agarose gel containing 1 μg/mL ethidium bromide (Sigma-Aldrich Pty Ltd) (results not shown). The genomic DNA concentration was standardised for all samples to 10 ng/μL before PCR amplification. These DNA samples then were sent to the Australian Genome Research Facility (AGRF, University of Queensland, Brisbane, QLD, Australia) for bacterial 16S rRNA gene sequencing using the Roche 454 platform.

### PCR amplification of the bacterial 16S rRNA gene

The PCR assay was performed at AGRF using a Forward 27 F primer (AGAGTTTGATCMTGGCTCAG) and a Reverse 519 R primer (GWATTACCGCGGCKGCTG)^41^, with fusion primer sequences for both the forward and reverse directions for each amplicon. Each primer sequence included the appropriate adapter sequence for Lib-L or Lib-A libraries followed by the key sequence of TCAG. This was followed by the unique “barcode” sequences for a GS FLX MID sequencing platform for forward and reverse directions; the barcode sequences were used subsequently to identify and distinguish each of the samples. Table 2 summaries the design structure of the fusion primers, and the 16S rRNA target sequence primers [Adapter]-[Key]-[MID barcode]-[Target primers].

PCR amplification was conducted in 384 well plates using 8 μL total volume per reaction. Each PCR reaction contained: 2 μL DNA template, 4 μL master mix containing Ampli Taq Gold (AmpliTAQ GOLD 360, Applied Biosystems, USA), and 2.25 μM of primers. The PCR cycling parameters were: 94 °C for 3 mins; followed by 34 cycles of 94 °C for 45 sec, 50 °C for 60 sec, 72 °C for 60 sec; a final extension step at 72 °C for 7 mins; and then the reactions were held at 10 °C. All amplicons were purified using a robotic liquid handler (Beckman Coulter 384 Biomek NX, CA, USA) and Agencourt AMPure (Beckman Coulter, CA, USA).

### Sample QC post PCR purification

The PCR amplicons were quantified using PicoGreen^®^ (Invitrogen, Mulgrave, VIC, Australia) to determine the DNA concentration and samples were normalised to the same molarity, then a qPCR reaction was performed to check each sample. The samples were re-normalised based on the qPCR copy number results. This double normalisation technique was effective at ensuring an even number of de-multiplexed reads post sequencing on the GSFLX.

### 16S rRNA gene sequencing (‘deep sequencing’)

Samples for bead-based sequencing were set up according to Roche’s standard Lib-L emPCR method for XLR70 chemistry on the GSFLX + instrument, emulsion PCR (emPCR) Method Manual – Lib-L MV GX FLX Titanium Series October 2009 (Rev. Jan 2010), Germany. Emulsions comprised 2 x medium volume, both at 0.35 copies per bead (cpb) to achieve recoveries of 18% and 20% (the recommended range is 5% to 20%).

Two million beads were sequenced in a half Pico titre plate in Roche-454 GS FLX amplicon sequencing platform (Roche, Castle Hill, NSW, Australia) according to Roche’s standard XLR70 protocol for the GSFLX + instrument. Subsequently, the quantified libraries were amplified in micro-reactors through emPCR followed by streptavidin bead enrichment and emulsion breaking. The beads attached to amplified DNA fragments were denatured with 1 N NaOH solution and annealed to a specific sequencing primer. All these steps and subsequent sequencing steps on the Roche-454 GS FLX amplicon sequencing platform were performed according to the Roche sequencing manual protocol. All sequence files were taken in fastq format then analysed using FASTQC. The FASTQC quality analysis tool assisted in determining the length of amplicons and its quality score distribution^42^. Standard bioinformatics quality control (QC) involved reviewing internal QC metrics and de-multiplexing the results as per the barcodes included in the pool.

### 454 bacterial 16S rRNA sequence analysis and statistics

The composition of species diversity in high-throughput amplicon sequencing data samples was analyzed using the Quantitative Insights Into Microbial Ecology (QIIME) software package version 1.7.0 (http://qiime.org/) for profiling bacterial 16S rRNA^43^. In order to analyse the sequence dataset using the QIIME software/protocol, the sequence dataset was first depleted of sequences with lengths below 250 bp but not higher than 550 bp^44^. The FASTQC length distribution is shown in the Supplementary Info Figure 1.

Sequences with more than six homopolymer runs were discarded and no barcode or primer mismatches were allowed. Low quality sequences with a phred score <25 (<Q25) and the reads from the forward primer were then selected and sample identities (breast-fed, formula-fed; 4, 6, or 8 weeks of age) were assigned based on barcode sequences. All barcodes and primer sequences were trimmed. The remaining high quality reads were then used to determine the diversity and richness of bacterial communities and the relative abundance based on operational taxonomic unit (OTU) using QIIME version 1.7.0.

Mean values of infant characteristics (gestational age, birth weight and age at the time of sampling) in breast-fed and formula-fed infants were analysed using an unpaired, two-tailed t-test. One-way analysis of variance (ANOVA) was performed to compare the relative abundance of phyla and genera in breast-fed and formula-fed babies. Regression analyses were employed for analysis of feeding mode over time (i.e. at 4, 6 and or 8 weeks of age). All p-values were calculated at 95% confidence intervals and statistical significance was accepted as p < 0.05.

### Total bacterial community composition

Bacterial communities were grouped into clusters (6101, in total) based on 3% (or 97% identity cut-off) sequence variation using UCLUST clustering algorithm^45^. Each cluster considered as an OTU and a representative OTU was selected to represent each cluster. Representative OTU sequence reads were aligned using QIIME based Nearest Alignment Space Termination (NAST) alignment. For taxonomy-based analysis, the Ribosomal database project (RDP) Naïve Bayesian classifier^44^ was used at a confidence threshold of 85% against the Greengenes (version 13_8) database. QIIME based composition analysis was used to quantify the total number of reads per each OTU and their relative abundance at phylum and genus levels. Bacterial OTUs assigned with one sequence read were removed prior to composition analysis. To account for different number of reads across individuals, raw abundance counts were normalised using total sum scaling method, which present the contribution of each bacterial taxa as a relative proposition to individual reads count^46^.

The Shannon diversity index was calculated as a measure of species richness and evenness of bacterial taxa within individual samples of breast-fed and formula-fed babies. The diversity between the samples (Beta diversity) was estimated using unweighted UniFrac distances^47^. Box plot graphs, cluster and heatmap diagrams were generated to illustrate bacterial community diversity and compositions using R project (ggplot2 package) for statistical computing (http://www.r-project.org) and Calypso web Online tool (http://bioinfo.qimr.edu.au/calypso). Phylogenetic trees were constructed using Randomised accelerated Maximum Likelihood (RaxML) HPC version 7.2.8^48^. Sequence reads were stored and managed using Filemaker Pro Advanced relational database management software (10.0 Ver.1) and diagrams were edited using Pages software on Mac OSX.

### Determination of ‘Bacteria_others’

Sequences from ‘Phylum-Unclassified’ bacteria identified by deep sequencing were selected from OTU clusters. In this study we focussed only on the OTUs with high abundance and occurrence. We excluded the OTUs with single read (singletons) and ones that appeared in less than 50% of samples. In total, 106 OTUs selected from 653 OTUs identified as ‘Bacteria_others’. The taxonomic identities of these ‘Bacteria_others’ OTUs were further explored using RDP naïve Bayesian classifier (16S bacteria training set 9) and the relative abundance of ‘Bacteria_others’ OTUs were shown together with predicted class levels taxonomic assignments. The bootstrap sequence similarity percentage of OTUs and their composition was described using a heat map diagram constructed using R project for statistical computing. In addition, we traced the source of origin of identified bacterial taxa. A phylogenetic was then constructed a using Randomised accelerated Maximum Likelihood (RaxML) HPC version 7.2.8 with 1000 bootstrap replications and General Time Reversal (GTR GAMMA) model.

## Additional Information

**How to cite this article**: Al-Shehri, S. S. *et al*. Deep sequencing of the 16S ribosomal RNA of the neonatal oral microbiome: a comparison of breast-fed and formula-fed infants. *Sci. Rep.* **6**, 38309; doi: 10.1038/srep38309 (2016).

**Publisher's note:** Springer Nature remains neutral with regard to jurisdictional claims in published maps and institutional affiliations.

## Supplementary Material

Supplementary Information

## Figures and Tables

**Table 1:** A comparison of the gestational age, birth weight, and age at the time of oral specimen collection, for breast-fed and formula-fed infants.

	Breast-fed			Formula-fed			*p* value
	Male	Female	Total	Male	Female	Total	
	12	14	26	5	7	12	
Gestational age (w)^1^							
Mean ± SD	40 ± 0.5	40 ± 1	40 ± 1	40 ± 2	39 ± 2	40 ± 2	0.3
Range	39–41	38–42		38–41	37–41		
Birth weight (g)							
Mean ± SD	3488 ± 316	3584 ± 570	3539 ± 464	3916 ± 599	3664 ± 227	3779 ± 432	0.2
Range	3054–4110	2700–4492		3182–4720	3490–4092		
Age (w) at collection^2^							
Mean ± SD	6 ± 1	6 ± 2	6 ± 2	7 ± 1	6 ± 2	6 ± 1	0.2
Range	4–8	4–8		6–8	4–8		

^1^w = weeks, at the time of delivery; term >37 weeks gestation.

^2^w = weeks, at the time of collection of oral specimen.

**Table 2:** The fusion primers design for 16S rRNA amplification, the fusion primer sequences contained [Adapter]-[Key]-[MID barcode]-[Target primers].

	Forward fusion primer sequence	Reverse fusion primer sequence
Adaptor	5′CCATCTCATCCCTGCGTGTCTCCGACC	5′CCTATCCCCTGTGTGCCTTGGCAGTC
Key	TCAG	TCAG
MID	Barcode sample specific	—
Target primer*	AGAGTTTGATCMTGGCTCAG	GWATTACCGCGGCKGCTG

^*^Base redundancies were included within the target primers (W = A + T, K = T + G, M = A + C).
