# Supplementary material for: Deep sequencing of the 16S ribosomal RNA of the neonatal oral microbiome: a comparison of breast-fed and formula-fed infants
Source: Sci Rep. 2016 Dec 6;6:38309. doi: 10.1038/srep38309 (PMC5138828; doi:10.1038/srep38309)

Deep sequencing of the 16S ribosomal RNA of the neonatal oral microbiome: a comparison of breast-fed and formula-fed infants

Authors
Al-Shehri SS
Sweeney EL
Cowley DM
Liley HG
Ranasinghe PD
Charles BG
Shaw PN
Vagenas D
Duley JA
Knox CL

Supplementary Table 1: Detailed sequences length distributions.

Length distribution for the first 36 samples			
Length	Raw	trimmed_primer_included	trimmed_primer_excluded
40	57	0	0
50	235	0	0
60	273	0	0
70	106	0	0
80	85	0	0
90	34	0	0
100	53	0	0
110	37	0	0
120	23	0	0
130	63	0	0
140	70	0	0
150	75	0	0
160	32	0	0
170	34	0	0
180	166	0	0
190	287	0	0
200	33	0	0
210	18	0	0
220	62	0	43
230	18	0	7
240	46	0	3
250	57	44	15
260	9	6	167
270	8	5	53
280	28	21	15
290	254	192	8
300	32	23	6
310	18	12	5
320	30	13	16
330	7	2	13
340	21	10	13
350	38	15	6
360	20	12	5
370	14	11	4
380	4	4	2
390	7	5	1
400	8	4	3
410	3	2	5
420	7	1	3

430	7	4	3
440	5	5	6
450	5	3	9
460	5	4	81
470	6	5	1598
480	21	10	111191
490	292	236	327156
500	2510	1913	34401
510	188585	150134	7667
520	371379	299523	452
530	31292	25404	0
540	6186	5214	0
550	698	120	0
560	184	0	0
570	56	0	0
580	20	0	0
590	9	0	0
600	3	0	0
610	3	0	0
620	2	0	0
630	2	0	0
640	3	0	0
650	1	0	0
660	3	0	0
670	3	0	0
680	1	0	0
690	1	0	0
700	2	0	0
710	1	0	0
720	3	0	0
730	0	0	0
740	2	0	0
750	3	0	0
760	2	0	0
770	0	0	0
780	3	0	0
790	1	0	0
800	1	0	0
810	1	0	0
820	2	0	0
830	0	0	0
840	2	0	0
850	1	0	0

860	1	0	0
870	1	0	0
880	0	0	0
890	0	0	0
900	1	0	0
910	0	0	0
920	0	0	0
930	0	0	0
940	0	0	0
950	1	0	0
960	1	0	0
970	0	0	0
980	0	0	0
990	0	0	0
1000	0	0	0
1010	0	0	0
1020	0	0	0
1030	0	0	0
1040	0	0	0
1050	0	0	0
1060	0	0	0
1070	0	0	0
1080	0	0	0
1090	0	0	0
1100	0	0	0
1110	0	0	0
1120	0	0	0
1130	0	0	0
1140	0	0	0
1150	0	0	0
1160	0	0	0
1170	0	0	0
1180	0	0	0
1190	1	0	0

	Raw		Trimmed_primer_excluded (This is the set of reads used for analysis)		
First 36 samples	519.79±23.52		492.97±8.33		
two(remaining samples)	535.03±33.75		498.18±5.45		
*First 43 samples includes only BF and FF samples					
two(remaining samples) includes one BF and one FF sample					

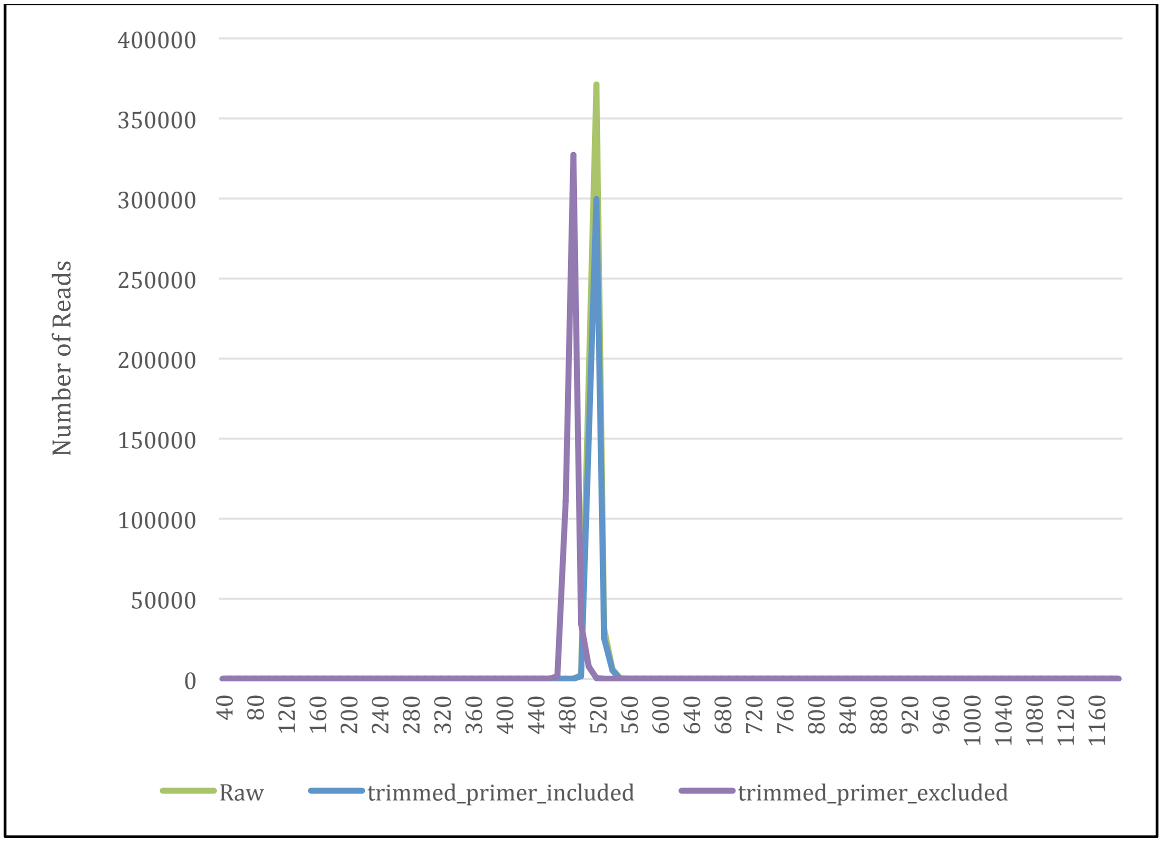

Supplementary Figure 1: The FASTQC sequences length distribution

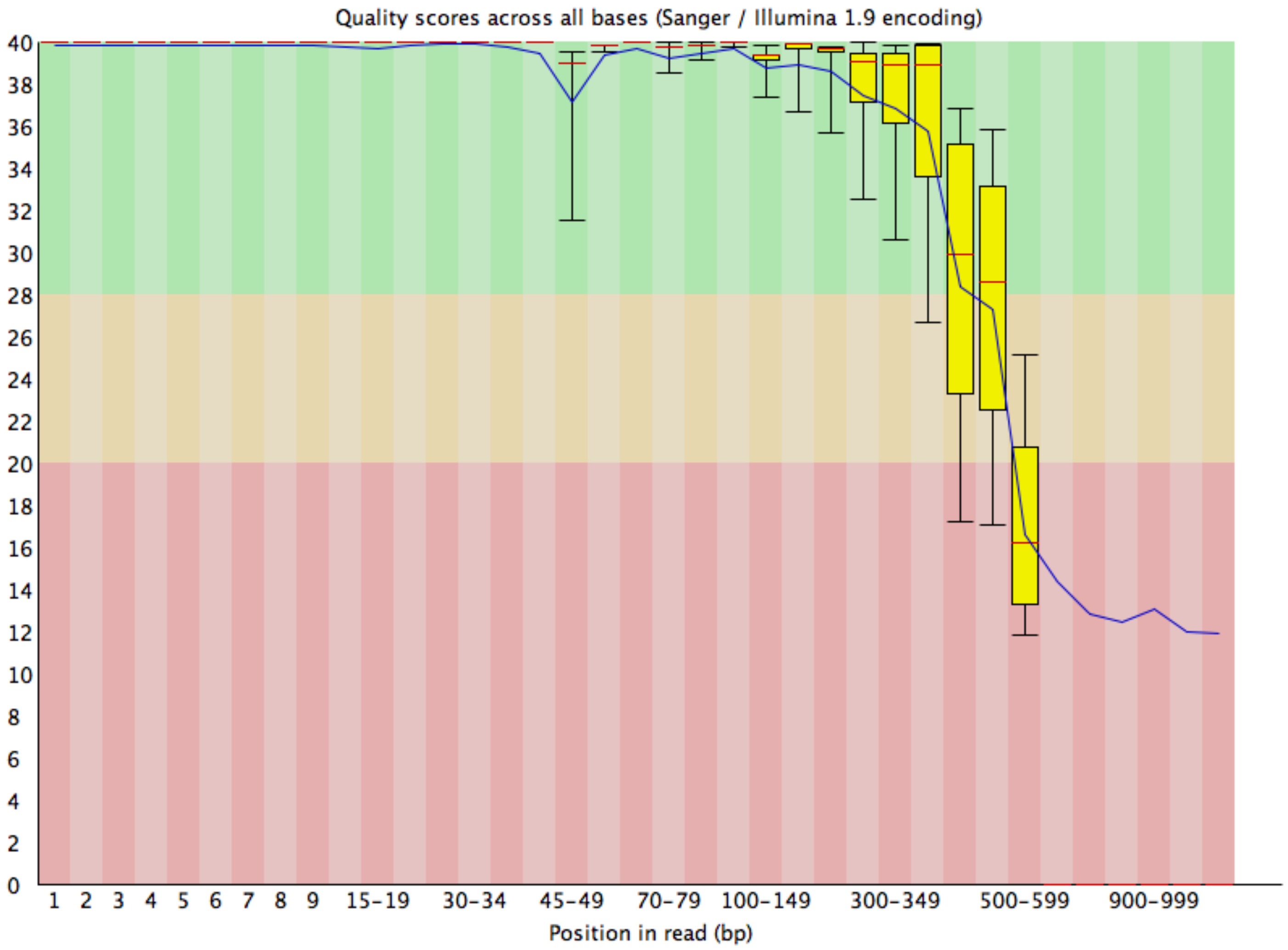

Supplement: Supplementary Information [file srep38309-s1.pdf]
